# Diagnostic Accuracy of Left Atrial/Left Atrial Appendage Thrombus in Patients with Atrial Fibrillation: A Systematic Review and Network Meta-Analysis

**DOI:** 10.31083/j.rcm2411334

**Published:** 2023-11-27

**Authors:** Ruirui Song, Fang Liu, Xiaojing Shi, Hongmei Gao, Jun Chen, Xuefeng Guo, Jian Huang

**Affiliations:** ^1^Cardiology, The Second Affiliated Hospital of Shandong University of Traditional Chinese Medicine, 250002 Jinan, Shandong, China

**Keywords:** atrial fibrillation, thrombus of LA/LAA, diagnosis, CMRI

## Abstract

**Background:**

This paper aimed to appraise the diagnostic precision of assorted methodologies to identify left atrial/left atrial appendage (LA/LAA) thrombus through a network meta-assessment.

**Methods:**

Methodologically, we conducted a comprehensive literature search across multiple databases. Utilizing the risk of bias tool from the Cochrane Collaboration, methodological quality of included studies was critically assessed and potential publication bias was examined via funnel plots. The subsequent data analysis was executed using Stata software, with the most efficacious diagnostic modalities being determined based on cumulative ranking curve (SUCRA) values.

**Results:**

We scrutinized a sum of 18 papers, comprising 4102 subjects and utilizing 10 different diagnostic techniques. The hierarchical results derived from the network meta-analysis indicated that in regards to sensitivity, the dual-source cardiac computed tomography (DSCT) was superior (with a SUCRA value of 71.7%), it was succeeded by 3-minute delayed cardiac computed tomography (CCT) (scoring 66.8%), which surpassed the transesophageal echocardiography (TEE) (holding a SUCRA value of 57.5%). In terms of specificity, DSCT was the best (SUCRA value of 84.3%), followed by three dimensional (3D) cardiac magnetic resonance imaging (3D-CMRI) (SUCRA value of 78.0%), which was better than TEE (SUCRA value of 66.6%). In terms of positive likelihood ratio (PLR), 6-minute delayed CCT (SUCRA value of 85.6%) was superior to 3-minute delayed CCT (SUCRA value of 80.1%), both of which were superior to TEE (SUCRA value of 69.1%). DSCT (SUCRA value of 89.3%) had the best negative likelihood ratio (NLR), while DSCT (SUCRA value of 79.9%) had the highest accuracy.

**Conclusions:**

This study demonstrated that DSCT outperformed TEE in sensitivity, specificity, NLR, and accuracy in identifying thrombus of LA/LAA among patients suffering from atrial fibrillation. Our conclusion is that DSCT is the best in diagnosing LA/LAA. In addition, 3D-CMRI and 3-minute delayed CCT are expected to replace TEE.

## 1. Introduction

In the spectrum of clinical arrhythmias, atrial fibrillation (AF) predominates. Around 59.7 million people worldwide have AF (including atrial flutter) as of 2019 [1]. AF has an increased all-cause mortality by 1.5-fold in men and 2-fold in women [2]. Currently, the treatment of AF consists mainly of drugs and catheter ablation. In the last decade, some antiarrhythmic drugs have been found to have a risk of causing arrhythmias [3, 4], and therefore have limitations in their clinical application. Catheter ablation offers significant advantages in maintaining sinus rhythm nevertheless, it doesn’t render an exception for the left atrial/left atrial appendage (LA/LAA) thrombus. More than 90% of the LA thrombus is present in the LAA, a special structure of the LA in which blood flow is slow and stagnant, leading to thrombus formation. The thrombus and emboli circulate through the blood stream to the cerebral arteries, blocking the blood supply to the brain and leading to ischemic cerebral infarction. The risk rates of ischemic stroke and systemic circulation artery embolism caused by AF are 1.92% and 0.24%, respectively. This results in a 20% mortality rate and a 60% disability rate [5], increases the number of cardiovascular diseases (49%), non-cardiovascular diseases (43%), and bleeding hospitalizations (8%) [6].

Currently, the main diagnostic methods for thrombus of LA/LAA include transesophageal echocardiography (TEE), cardiac magnetic resonance imaging (CMRI), multidetector computed tomography cardiac computed tomography, dual-source cardiac computed tomography (DSCT), computed tomography of the heart delayed, as well as cardiac computed tomography angiography (CCTA). However, there is minimal relevant research to determine the overall diagnostic efficacy of these various methods. TEE is decisive for determining LA/LAA thrombosis [7, 8]. Because TEE has certain complications and some patients cannot tolerate it because of Esophageal stricture, esophageal ulcer, anesthetic allergy/hypertension. Therefore, it is necessary to find alternative detection methods for TEE for TEE [9]. We use network meta-analysis (NMA) to make a comparison of the diagnostic result between various thrombus of LA/LAA detection techniques in order to offer solid suggestions for patients and clinicians.

## 2. Materials and Methods

### 2.1 Registration

This study using network meta-analysis (NMA) has been registered on the INPLASY-International Platform, Invoice Number: 2022120041.

### 2.2 Search Strategy

As of September 2022, the literature was retrieved using PubMed, EMBASE, an electronic database of Cochrane Controlled Trials, WOS (Web of Science), as well as other databases. The PICOS ( Patient, Intervention, Control ,Outcome and Study design) tool served as the foundation for the investigators’ search strategy: (P) Patients with atrial fibrillation who are receiving cardiac radiofrequency ablation, electrical cardioversion/cardiac evaluation for other reasons; (I) Interventional procedures include CMRI, cardiac computed tomography (CCT), TEE, transthoracic echocardiography (TTE), and other diagnostic modalities. (C) Control: Within one month, all patients underwent TEE examinations. (O) LAA/LA thrombus; (S) Study design: observational test. The search approach is shown in **Supplementary Table 1**.

### 2.3 Inclusion Criteria

(1) The investigators’ search strategy was carried out using the PICOS tool; (2) Methods: screening tools including TEE, and at least one diagnostic method; (3) Studies including the following metrics: TP (True positive), TN (True Negative), FP (False Positive), FN (False Negative), Se (Sensitivity), Sp (Specificity), Sr (Accuracy), NLR (Negative likelihood ratio). Furthermore, In the absence of positive likelihood ratio (PLR), NLR, TP, TN, FP, FN, calculations are made based on known variables (Se and Sp).

### 2.4 Exclusion Criteria

(1) Research without complete data; (2) Research lacking clear inclusion criteria; (3) Non-diagnostic pilot studies (including randomized controlled trials, animal studies, protocols, meeting summaries, case reports/letters).

### 2.5 Data Extraction

Ruirui Song and Jun Chen are responsible for searching the literature and importing the search results into file manager EndNote software (version 20.2.1, Bld 1574, Thomson ResearchSoft, USTC, USA). After Ruirui Song eliminated the duplicate literature, Ruirui Song, Jun Chen, Jian Huang and Xiaojing Shi screened the literature by reading the title, abstract and full text, and finally got the included literature after discussion and communication. Xuefeng Guo and Hongmei Gao extracted data from the included literature, respectively. If there was any doubt, they agreed on their opinions after consultation. Main extracted information: Author, Year, Country, Reference standard, Diagnostic method and main indicators Se, Sp, PLR, NLR, TP, FP, FN and TN.

### 2.6 Literature Quality Assessment

Hongmei Gao and Xiaojing Shi respectively used QUADAS-2 (Quality Assessment of Diagnostic Accuracy Studies) diagnostic accuracy research quality evaluation software [10] to determine the quality of the selected literature, discussed and unified the evaluation results. The scale evaluation included bias risk assessment and clinical applicability assessment. Bias risk is defined as “low”, “high”/“uncertain”. Discuss and resolve any disagreement with Fang Liu during quality evaluation.

### 2.7 Analysis of Data

In studies with different diagnostic methods used as interventions, all variables were continuous and represented by the mean and standard deviation. The study of continuous variable will take the mean differences (MD equals Mean difference between gold standard TEE and other diagnostic methods, calculated using the same scale), 95% confidence interval (CI), and analysis. Because there are differences among various research, the random effects model was chosen to analyze [11].

Stata software (version 15.1, StataCorp LLC, College Station, TX, USA) was recruited to perform mesh meta-analysis using a Bayesian framework based on the PRISMA NMA User Manual [12, 13]. Data were inputted into Stata15.1 software and *p*-values were obtained. Node method was used to quantify the consistency of the included study. If the *p*-value was above 0.05, it passed the consistency test [14].

Stata15.1 software was used to draw the network diagram, forest diagram and funnel diagram of LA/LAA with various diagnostic tools. Every node marks a type of diagnostic way, and the lines banding the nodes represent a straightforward, positive comparison between other diagnostic ways. The larger the node, the thicker the line, the more the number of studies, and vice versa [15].

Based on Bayesian method, the cumulative ranking curve (SUCRA) values can accurately represent the percentage of different diagnostic methods and intuitively display the performance of each diagnostic method. Through ranking, the most efficient diagnostic way can be obtained. A high SUCRA value indicates a high diagnostic performance rating for this diagnostic method [16]. The network funnel plot can show whether there is publication bias in the included literature, and the symmetry criterion is used for visual examination. If the funnel plot is asymmetrical, publication bias is likely to occur [17].

## 3. Results

### 3.1 Study Search

**Fig. 1.**
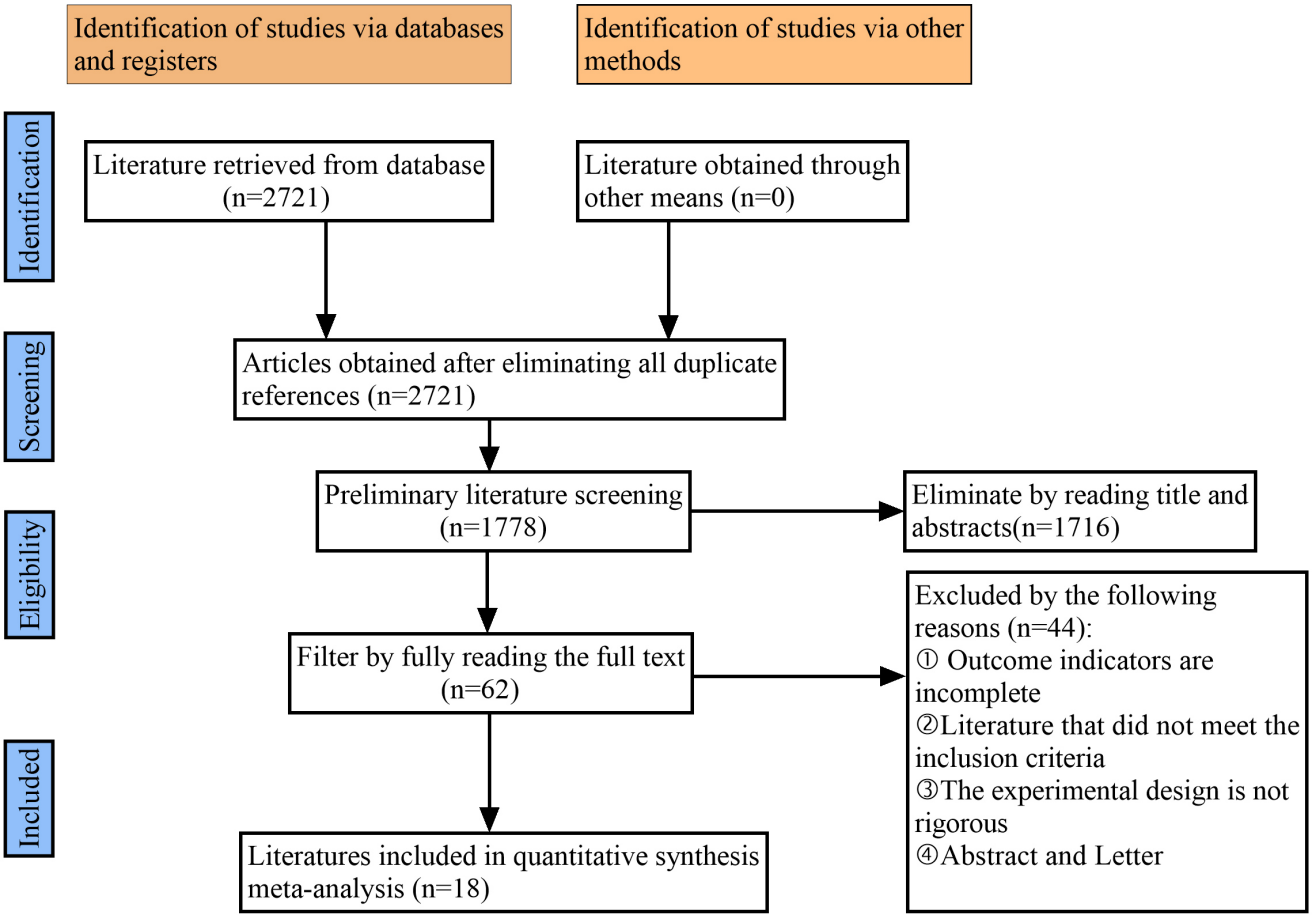
**Flow diagram of literature selection**.

2721 articles were retrieved. After excluding duplicate entries, 1778 articles remained. Then, by browsing the title, abstract and full text, 62 papers were selected from the 1716 papers, and further excluded literature such as unrigorous research design, imperfect outcome indicators and abstracts. Finally, 18 articles [18, 19, 20, 21, 22, 23, 24, 25, 26, 27, 28, 29, 30, 31, 32, 33, 34, 35] were included; flow diagram of references choice. Fig. 1 shows the detailed literature screening process.

### 3.2 Features of the Selected Studies

This study included 10 diagnostic tests and 4102 patients. TEE was the gold standard, with non-delayed CCT (4 studies) [18, 19, 20, 21], 1-minute delayed CCT (3 studies) [20, 21, 22], 3-minute delayed CCT (2 studies) [21, 22], 6-minute delayed CCT (1 study) [21], two dimensional (2D)-CMRI (1 study) [23], DSCT (2 studies) [24, 25], multidetector computed tomography (MDCT) (8 studies) [26, 27, 28, 29, 30, 31, 32, 33], three dimensional (3D)-TEE (1 investigation) [34], 3D-CMRI (1 investigation) [23], and CCTA (1 study) [35]. The features and baseline of the selected studies are demonstrated in Tables 1,2 (Ref. [18, 19, 20, 21, 22, 23, 24, 25, 26, 27, 28, 29, 30, 31, 32, 33, 34, 35]).

**Table 1.** **Baseline information of the meta-analysis**.

Author	Year	Country	Age (years) (mean ± SD)	Total/Female	Study design
Budoff MJ, *et al*. [35]	2014	California	64	84/16	retrospective
Dorenkamp M, *et al*. [26]	2013	Germany	62 ± 10	329/115	prospective
Hioki M, *et al*. [27]	2016	Japan	55.2 ± 10.7	459/40	prospective
Ikegami Y, *et al*. [18]	2017	Japan	69 (61–72)	95/77	retrospective
Kantarci M, *et al*. [28]	2019	Turkey	60	53/22	prospective
Kapa S, *et al*. [24]	2010	USA	59	255/56	retrospective
Kottmaier M, *et al*. [25]	2019	Germany	60 ± 10	622/193	prospective
Li XN, *et al*. [22]	2022	China	58.3 ± 12.2	329/102	retrospective
Martinez MW, *et al*. [29]	2009	Minnesota	56 ± 10	402/94	retrospective
Mohrs OK, *et al * [23]	2006	Germany	64 ± 10	23/–	prospective
Munir S, *et al*. [19]	2015	Canada	59.4 ± 9.5	51/13	retrospective
Patel A, *et al*. [30]	2008	New York	56.1 ± 10.3	72/22	retrospective
Sawit ST, *et al*. [20]	2012	New York	59.5 ± 12.4	70/19	prospective
Singh NK, *et al*. [31]	2009	Illinois	64 ± 10.3	51/14	retrospective
Spagnolo P, *et al * [21]	2021	Italy	59 ± 11	260/61	prospective
Squara F, *et al*. [34]	2018	France	76 (71–77)	104/42	prospective
Yasuoka R, *et al*. [32]	2017	Japan	64 ± 10	60/12	retrospective
Zhai Z, *et al*. [33]	2018	China	55 ± 11	783/231	retrospective

SD, standard deviation.

**Table 2.** **Traits of the researches selected in the meta-analysis**.

Authors	Reference standard	Diagnostic method	Total	Se	Sp	NPV	PPV	Accuracy
Budoff MJ, *et al*. [35]	TEE	CCTA	84	100%	77.90%	100%	51.60%	77.90%
Dorenkamp M, *et al*. [26]	TEE	MDCT	329	29%	98%	98%	20%	27%
Hioki M, *et al*. [27]	TEE	MDCT	459	100%	91%	100%	17.65%	91%
Ikegami Y, *et al*. [18]	TEE	non-delayed CCT	95	100%	81%	100%	40.70%	81%
Kantarci M, *et al*. [28]	TEE	MDCT	53	100%	100%	100%	100%	100%
Kapa S, *et al*. [24]	TEE	DSCT	255	100%	88%	100%	12.12%	100%
Kottmaier M, *et al*. [25]	TEE	DSCT	622	100%	89.20%	100%	4.30%	89.20%
Li XN, *et al*. [22]	TEE	1-minute delayed CCT	329	100%	93%	100%	57%	93%
		3-minute delayed CCT	329	100%	100%	100%	100%	100%
Martinez MW, *et al*. [29]	TEE	MDCT	402	100%	92%	100%	23%	92%
Mohrs OK, *et al*. [23]	TEE	2D-CMR	23	47%	50%	25%	73%	3%
		3D-CMR	23	35%	67%	27%	75%	2%
Munir S, *et al*. [19]	TEE	non-delayed CCT	51	0%	88%	100%	0%	12%
Patel A, *et al*. [30]	TEE	MDCT	72	100%	72.20%	100%	28.60%	72.20%
Sawit ST, *et al*. [20]	TEE	non-delayed CCT	70	100%	83.80%	100%	15%	83.80%
		1-minute delayed CCT	70	100%	100%	100%	100%	100%
Singh NK, *et al*. [31]	TEE	MDCT	51	100%	95.90%	100%	50%	95.90%
Spagnolo P, *et al * [21]	TEE	non-delayed CCT	260	100%	79%	100%	16%	79%
		1-minute delayed CCT	260	100%	98%	100%	67%	98%
		3-minute delayed CCT	260	100%	99%	100%	83%	99%
		6-minute delayed CCT	260	100%	100%	100%	100%	100%
Squara F, *et al*. [34]	TEE	3D-TEE	104	100%	99%	100%	89%	99%
Yasuoka R, *et al*. [32]	TEE	MDCT	60	90%	84%	100%	0%	74%
Zhai Z, *et al*. [33]	TEE	MDCT	783	100%	95.74%	100%	19.51%	96%

TEE, transesophageal echocardiography; CCTA, cardiac computed tomography angiography; MDCT, multidetector computed tomography; CCT, cardiac computed tomography; DSCT, dual-source cardiac computed tomography; 2D-CMR, 2D-cardiac magnetic resonance imaging; 3D-CMR, 3D-cardiac magnetic resonance imaging; Se, sensitivity; Sp, specificity; NPV, negative predictive value; PPV, positive predictive value; 2D, two dimensional; 3D, three dimensional.

### 3.3 Quality Evaluation of Selected Researches

**Fig. 2.**
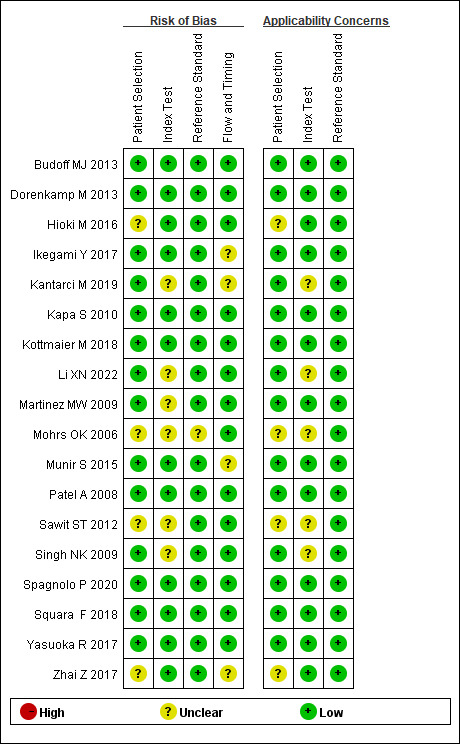
**Risk of bias and adaptability concerns summary**.

**Fig. 3.**
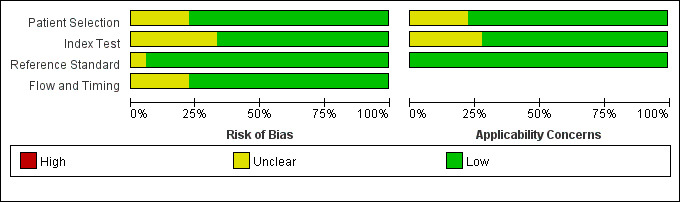
**Risk of bias and applicability concerns chart**.

The 18 studies (Ref. [17, 18, 19, 20, 21, 22, 23, 24, 25, 26, 27, 28, 29, 30, 31, 32, 34, 35]) included underwent network meta-analysis using STATA 15.1, and two researchers used QUADAS-2 to evaluate the study’s quality, deviation risk, and applicability. Where disagreements existed, they were discussed/resolved by a third party. The overall quality of the article was satisfactory: 8 studies [21, 24, 25, 26, 30, 32, 34, 35] were low-risk studies, and the remaining 10 studies [18, 19, 20, 22, 23, 27, 28, 29, 31, 33] were medium-risk. Fourteen studies [18, 19, 20, 21, 22, 23, 24, 25, 26, 28, 29, 31, 32, 33] described examiner blinding. However, due to the different diagnostic methods used in these studies, it is difficult to blind both patients and diagnosers at the same time, but most diagnosers in these studies are unaware (Figs. 2,3).

### 3.4 Network Meta-Analysis

**Fig. 4.**
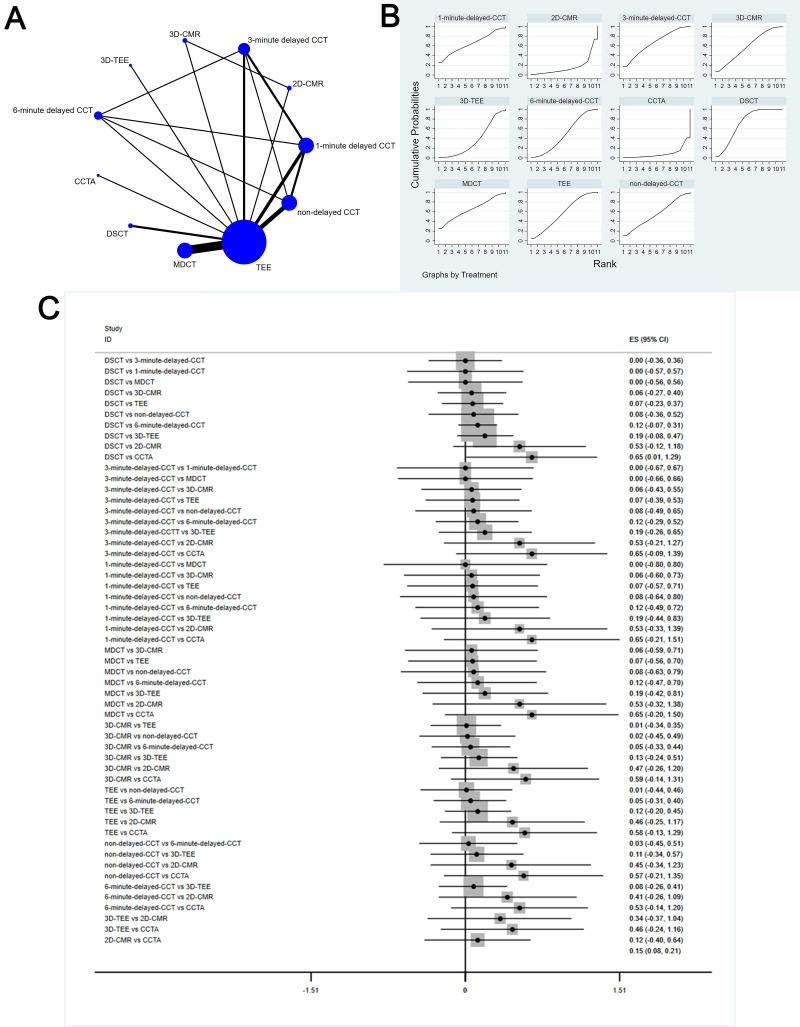
**Comparison of sensitivity of different diagnostic methods**. (A) NMA figure for Se. (B) SUCRA plot for Se. (C) Forest map for Se. TEE, transesophageal echocardiography; CCTA, cardiac computed tomography angiography; MDCT, multidetector computed tomography; CCT, cardiac computed tomography; DSCT, dual-source cardiac computed tomography; 2D-CMR, 2D-cardiac magnetic resonance imaging; 3D-CMR, 3D-cardiac magnetic resonance imaging; Se, sensitivity; NMA, network meta-analysis; SUCRA, surface under the cumulative ranking curve; 2D, two dimensional; 3D, three dimensional; CI, confidence interval; ES indicates effect size.

**Fig. 5.**
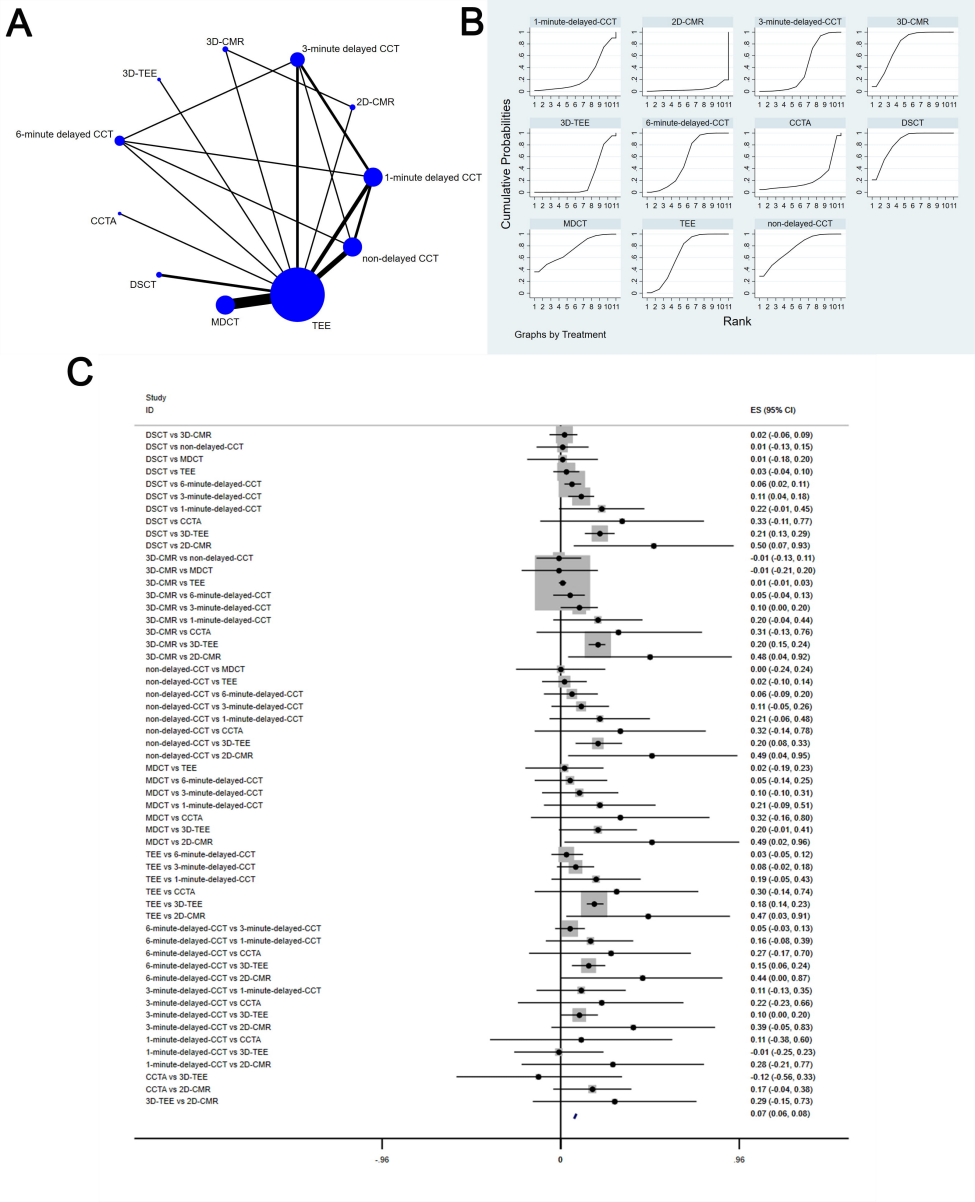
**Comparison of specificity of different diagnostic methods**. (A) NMA figure for Sp. (B) SUCRA plot for Sp. (C) Forest map for Sp. TEE, transesophageal echocardiography; CCTA, cardiac computed tomography angiography; MDCT, multidetector computed tomography; CCT, cardiac computed tomography; DSCT, dual-source cardiac computed tomography; 2D-CMR, 2D-cardiac magnetic resonance imaging; 3D-CMR, 3D-cardiac magnetic resonance imaging; NMA, network meta-analysis; Sp, specificity; SUCRA, surface under the cumulative ranking curve; 2D, two dimensional; 3D, three dimensional; CI, confidence interval; ES indicates effect size.

**Fig. 6.**
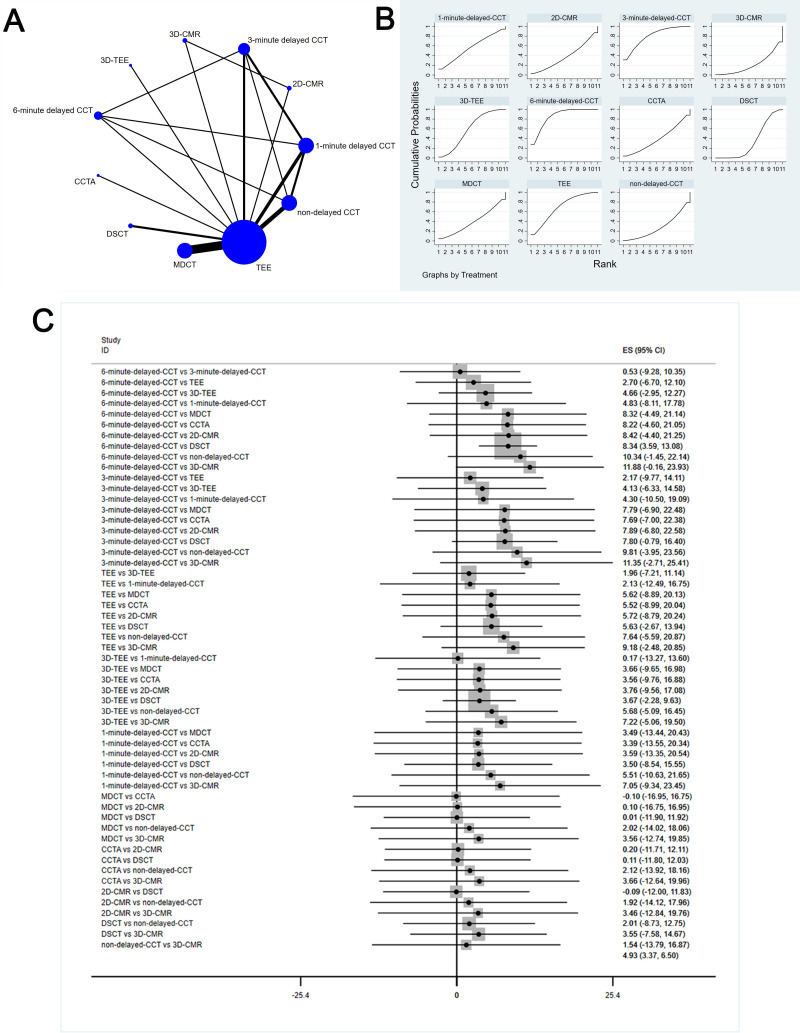
**Comparison of positive likelihood ratio of different diagnostic methods**. (A) NMA figure for PLR. (B) SUCRA plot for PLR. (C) Forest map for PLR. TEE, transesophageal echocardiography; CCTA, cardiac computed tomography angiography; MDCT, multidetector computed tomography; CCT, cardiac computed tomography; DSCT, dual-source cardiac computed tomography; 2D-CMR, 2D-cardiac magnetic resonance imaging; 3D-CMR, 3D-cardiac magnetic resonance imaging; NMA, network meta-analysis; PLR, positive likelihood ratio; SUCRA, surface under the cumulative ranking curve; 2D, two dimensional; 3D, three dimensional; CI, confidence interval; ES indicates effect size.

**Fig. 7.**
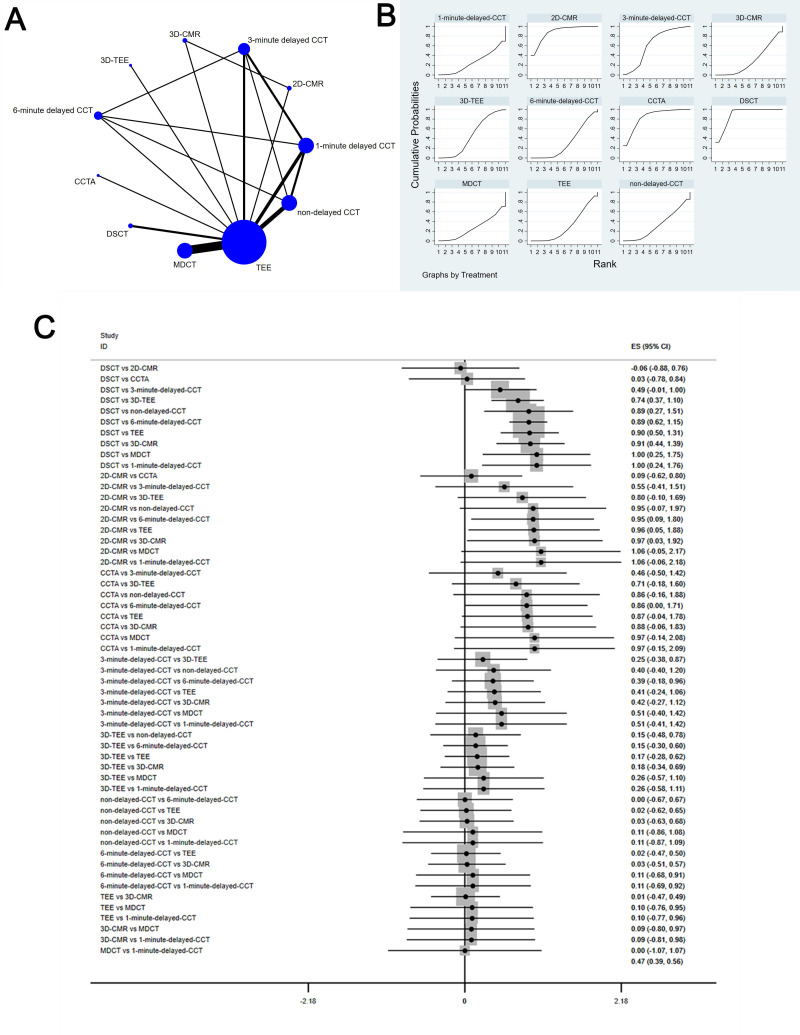
**Comparison of negative likelihood ratio of different diagnostic methods**. (A) NMA figure for NLR. (B) SUCRA plot for NLR. (C) Forest map for NLR. TEE, transesophageal echocardiography; CCTA, cardiac computed tomography angiography; MDCT, multidetector computed tomography; CCT, cardiac computed tomography; DSCT, dual-source cardiac computed tomography; 2D-CMR, 2D-cardiac magnetic resonance imaging; 3D-CMR, 3D-cardiac magnetic resonance imaging; NMA, network meta-analysis; SUCRA, surface under the cumulative ranking curve; NLR, negative likelihood ratio; 2D, two dimensional; 3D, three dimensional; CI, confidence interval; ES indicates effect size.

**Fig. 8.**
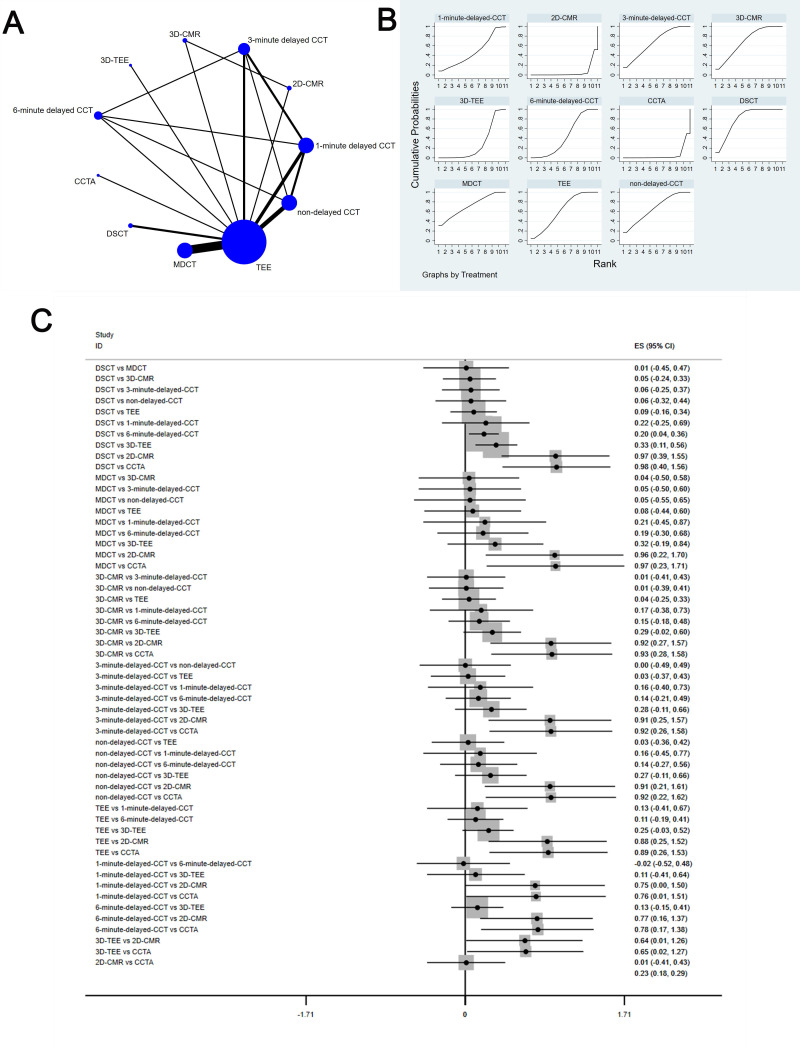
**Comparison of accuracy of different diagnostic methods**. (A) NMA for Accuracy. (B) SUCRA plot for Accuracy. (C) Forest map for Accuracy. TEE, transesophageal echocardiography; CCTA, cardiac computed tomography angiography; MDCT, multidetector computed tomography; CCT, cardiac computed tomography; DSCT, dual-source cardiac computed tomography; 2D-CMR, 2D-cardiac magnetic resonance imaging; 3D-CMR, 3D-cardiac magnetic resonance imaging; NMA, network meta-analysis; SUCRA, surface under the cumulative ranking curve; 2D, two dimensional; 3D, three dimensional; CI, confidence interval; ES indicates effect size.

The complete NMA is showed in Figs. 4,5,6,7,8A.

#### 3.4.1 Sensitivity

This study showed a big difference in the sensitivity of DSCT [MD equals 0.65, 95% confidence interval: (0.01, 1.29)] compared to CCTA.

The SUCRA values for DSCT (71.7%) > 3-minute delayed CCT (66.8%) > 1-minute delayed CCT (64.7%) > MDCT (64.7%) > 3D-CMRI (58.9%) > TEE (57.5%) > non-delayed CCT (55.2%) > 6-minute delayed CCT (48.6%) > 3D-TEE (36.3%) > 2D-CMRI (16.8) > CCTA (8.8%) are shown in Fig. 4B; Fig. 4C shows the Forest map for Se which comparison between these different diagnostic measures.

#### 3.4.2 Specificity

This study showed that 6-minute delayed CCT [MD equals 0.06, 95% confidence interval: (0.02, 0.11)], 3-minute delayed CCT [MD equals 0.11, 95% confidence interval: (0.04, 0.18)], 3D-TEE [MD equals 0.21, 95% confidence interval: (0.13, 0.29)], and 2D-CMRI [MD equals 0.50, 95% confidence interval: (0.07, 0.93)] have significant differences in specificity compared with DSCT; compared to 2D-CMRI, TEE [MD equals 0.47, 95% confidence interval: (0.03, 0.91)], MDCT [MD equals 0.50, 95% confidence interval: (0.07, 0.93)], non-delayed CCT [MD equals 0.49, 95% confidence interval: (0.04, 0.95)], and 3D-CMRI [MD equals 0.48, 95% confidence interval: (0.04, 0.92)] have significant differences in specificity; compared to 3D-TEE, 3D-CMRI [MD equals 0.20, 95% confidence interval: (0.15, 0.24)], non-delayed CCT [MD equals 0.2, 95% confidence interval: (0.08, 0.33)], TEE [MD equals 0.18, 95% confidence interval: (0.4, 0.23)], and 6-minute delayed CCT [MD equals 0.15, 95% confidence interval: (0.06, 0.24)] have pronounced differences in specificity.

The SUCRA values for DSCT (84.3%) > 3D-CMRI (78.0%) > non-delayed CCT (76.8%) > MDCT (74.3%) > TEE (66.6%) > 6-minute delayed CCT (55.2%) > 3-minute delayed CCT (40.2%) > 1-minute delayed CCT (26%) > CCTA (22.5%) > 3D-TEE (21.9%) > 2D-CMRI (4.3%) are shown in Fig. 5B; Fig. 5C shows the Forest map for Sp which comparison between these different diagnostic measures.

#### 3.4.3 Positive Likelihood Ratio

This study showed a significant difference in PLR for DSCT [MD equals 8.34, 95% confidence interval: (3.59, 13.08)] compared to 6-minute delayed CCT.

The SUCRA values for 6-minute delayed CCT (85.6%) > 3-minute delayed CCT (80.1%) > TEE (69.1%) > 3D-TEE (59.3%) > 1-minute delayed CCT (26.0%) > MDCT (39.3%) > CCTA (39.0%) > 2D-CMRI (37.8%) > DSCT (34.3) > non-delayed CCT (28.2%) > 3D-CMRI (21.3%) are shown in Fig. 6B; Fig. 6C shows the Forest map for Sp which comparison between these different diagnostic measures.

#### 3.4.4 Negative Likelihood Ratio

The network meta-analysis results demonstrated that 3D-TEE [MD equals 0.74,95% confidence interval: (0.37, 1.1)], non-delayed CCT [MD equals 0.89, 95% confidence interval: (0.27, 1.51)], 6-minute delayed CCT [MD equals 0.89, 95% confidence interval: (0.62, 1.15)], TEE [MD equals 0.9, 95% confidence interval: (0.5, 1.31)], 3D-CMRI [MD equals 0.91, 95% confidence interval: (0.44, 1.39)], MDCT [MD equals 1, 95% confidence interval: (0.25, 1.75)], and 1-minute delayed CCT [MD equals 1, 95% confidence interval: (0.24, 1.76)] have significant differences in NLR compared with DSCT; compared to 2D-CMRI, 6-minute delayed CCT [MD equals 0.95, 95% confidence interval: (0.09, 1.8)], TEE [MD equals 0.96, 95% confidence interval: (0.05, 1.88)], and 3D-CMRI [MD equals 0.97, 95% confidence interval: (0.03, 1.92)] have significant differences in NLR.

The SUCRA values for DSCT (89.3%) > 2D-CMRI (88.3%) > CCTA (84%) > 3-minute delayed CCT (63.8%) > 3D-TEE (46.7%) > non-delayed CCT (32.8%) > 6-minute delayed CCT (31.9%) > TEE (30.4%) > 3D-CMRI (29.6%) > MDCT (26.6%) > 1-minute delayed CCT (26.5%) are as shown in Fig. 7B; Fig. 7C shows the Forest map for NLR which comparison between these different diagnostic measures.

#### 3.4.5 Accuracy

This study showed that compared to DSCT, 3D-TEE [MD equals 0.33, 95% confidence interval: (0.11, 0.56)], 6-minute delayed CCT [MD equals 0.2, 95% confidence interval: (0.04, 0.36)], 2D-CMRI [MD equals 0.97, 95% confidence interval: (0.39, 1.55)], as well as CCTA [MD equals 0.98, 95% confidence interval: (0.4, 1.56)] have significant differences in accuracy; compared to 2D-CMRI, 3D-TEE [MD equals 0.64, 95% confidence interval: (0.01, 1.26)], TEE [MD equals 0.88, 95% confidence interval: (0.25, 1.52)], non-delayed CCT [MD equals 0.91, 95% confidence interval: (0.21, 1.61)], 3-minute delayed CCT [MD equals 0.91, 95% confidence interval: (0.25, 1.57)], 6-minute delayed CCT [MD equals 0.77, 95% confidence interval: (0.06, 1.37)], and 3D-CMRI [MD equals 0.92, 95% confidence interval: (0.27, 1.57)] have significant differences in accuracy; compared to CCTA, 3D-CMRI [MD equals 0.93, 95% confidence interval: (0.28, 1.58)], 3-minute delayed CCT [MD equals 0.92, 95% confidence interval: (0.26, 1.58)], non-delayed CCT [MD equals 0.92, 95% confidence interval: (0.22, 1.62)], TEE [MD equals 0.89, 95% confidence interval: (0.26, 1.53)], 1-minute delayed CCT [MD equals 0.76, 95% confidence interval: (0.01, 1.51)], 3D-TEE [MD equals 0.65, 95% confidence interval: (0.02, 1.27)], and 6-minute delayed CCT [MD equals 0.78, 95% confidence interval: (0.17, 1.38)] have significant differences in accuracy.

The SUCRA values for DSCT (79.9%) > MDCT (72.0%) > 3D-CMRI (69.5%) > 3-minute delayed CCT (67.5%) > non-delayed CCT (67.0%) > TEE (63.0%) > 1-minute delayed CCT (47.2%) > 6-minute delayed CCT (44.6%) > 3D-TEE (27.9%) > 2D-CMRI (5.9%) > CCTA (5.6%) are shown in Fig. 8B; Fig. 8C shows the comparison between these different diagnostic measures.

### 3.5 Publication Bias Examination

**Fig. 9.**
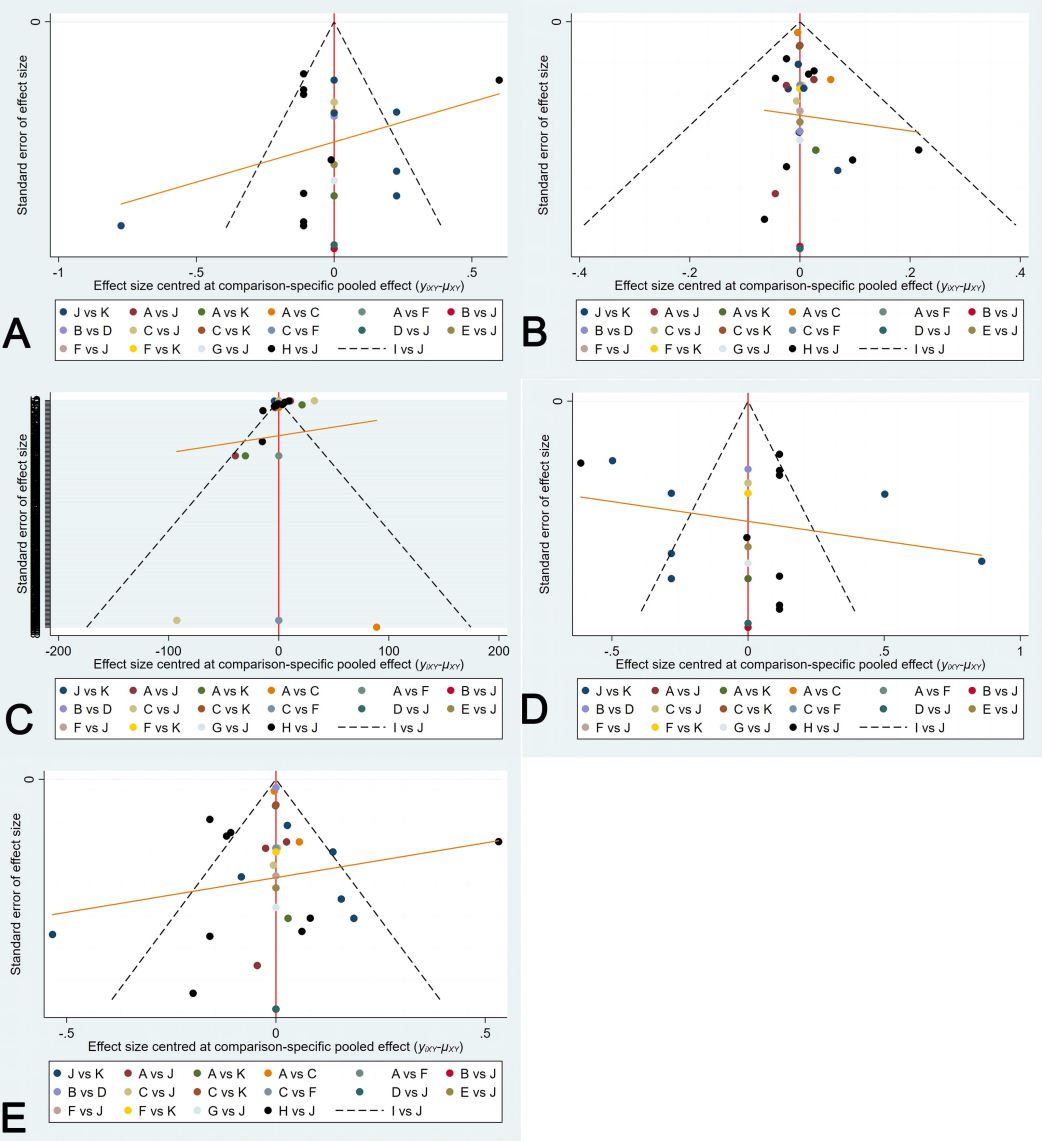
**The fuel plots of network meta-analysis**. (A) Funnel plot for Se. (B) Funnel plot for Sp. (C) Funnel plot for PLR. (D) Funnel plot for NLR. (E) Funnel plot for Accuracy. Se, sensitivity; Sp, specificity; PLR, positive likelihood ratio; NLR, negative likelihood ratio.

Funnel plots were drawn for Se, Sp, PLR, NLR and Accuracy to determine possible publication bias. Visually, the overall publication bias of the literature was small. The details are shown in Fig. 9.

## 4. Discussion 

Among various diagnostic methods for LA/LAA thrombosis in clinical practice, TEE is still the gold standard for recommendations. In this study, network meta-analysis was performed on different diagnostic methods to compare the diagnostic value of different diagnostic techniques for LA/LAA thrombosis detection, so as to provide evidence for clinical application. Our study participants included 18 articles (Ref. [17, 18, 19, 20, 21, 22, 23, 24, 25, 26, 27, 28, 29, 30, 31, 32, 34, 35]), including 4102 patients and 10 kinds of diagnostic methods. The ranking results of the NMA demonstrated that in terms of sensitivity, DSCT is the best (SUCRA value of 71.7%), followed by 3-minute delayed CCT (66.8%), which was better than TEE (SUCRA value of 57.5%). In terms of specificity, DSCT is the best (SUCRA value of 84.3%), followed by 3D-CMRI (SUCRA value of 78.0%), which was better than TEE (SUCRA value of 66.6%). In terms of PLR, 6-minute delayed CCT (SUCRA value of 85.6%) was superior to 3-minute delayed CCT (SUCRA value of 80.1%), both of which were superior to TEE (SUCRA value of 69.1%). DSCT (SUCRA value of 89.3%) had the highest NLR, while DSCT (SUCRA value of 79.9%) had the highest accuracy. This study demonstrated that besides the gold standard, DSCT was the best in diagnosing LA/LAA thrombosis. In addition, 3D-CMRI and 3-minute delayed CCT are expected to replace TEE. Through the literature review, several meta-analyses were conducted on two/ more methods for diagnosing thrombus of LA/LAA. However, this study conducted a comprehensive network meta-analysis of these diagnostic methods.

Because of LAA’s complex structure and physiological characteristics, finding a substitution to TEE for the diagnosis of LAA thrombosis is challenging. CMRI is a safe and non-invasive examination method that does not require sedation, iodized contrast agents/ionizing radiation. A meta-analysis showed that [36] CMRI has a sensitivity of 44–100%, a specificity of 67–100%, an active forecast result of 50–100%, a passive forecast result of 29–100%, and the SUCRA value is 0.93 in the diagnosis of thrombus of LA/LAA. This confirms that CMRI is a reliable diagnostic method to detect thrombus of LA/LAA. It was shown that CMRI can evaluate thrombus of LA/LAA among patients suffering from non-rheumatic atrial fibrillation as well as patients who once had a stroke [37]. They concluded that the TEE and CMRI 100% of consistency in terms of detection LAA blood clots. Rathi *et al*. [38] showed that CMRI detection of LAA/LA thrombus is specific to TEE and can detect intracardiac thrombus other than the LAA/LA. In addition, 3D-CMRI can clearly display pulmonary vein anatomy, identify structural variations such as pulmonary vein stenosis, and provide additional cardiac electrophysiological information such as LA fibrosis [39]. CMRI is safe and non-invasive for the diagnosis of thrombus of LA/LAA in patients with atrial fibrillation, but compared with TEE, CMRI has high cost and high technical requirements, which hinders its wide clinical development.

A computer tomography (CT) meta-analysis consisting of 9 researches [40] revealed that the average sensitivity as well as specificity of CCT within diagnosing thrombus of LA/LAA in patients with AF were 81% (95% confidence interval: 70–90%) and 90% (95% confidence interval: 88–91%), respectively. Five types of CT were included in this study, including MDCT, DSCT, CCTA, delayed CT and non-delayed CT. The outcomes demonstrated that DSCT was better than TEE in terms of sensitivity, specificity, NLR and accuracy. DSCT is the latest technology emerging in the development process of CT. Due to the fact that respiration, heartbeat, and AF can cause PV and LA movements, which affects the detection ability of conventional CT, AF is considered a contraindication for single source CT cardiac angiography [41]. DSCT improves the time resolution of synchronous cardiac scanning and proves that DSCT can successfully image the heart and coronary arteries at high heart rates and complex rhythms. In addition, the combination of DSCT and electrocardiogram gating technology quickly covers the heart, resulting in a reduction in radiation exposure compared to standard MDCT [42, 43]. Due to the shortened examination time and reduced use of contrast agents, the safety for renal insufficiency patients has improved [44]. A meta-analysis suggests that low-dose angiography also maintains the same accuracy [27]. With the continuous development of technology, DSCT needs to further improve image reconstruction and cross scattering radiation technology, providing greater utilization for clinical and academic applications.

## 5. Advantages and Limitation

There are numerous methods to diagnose LAA/LA thrombus, and several meta-analyses of CT and CMRI have been performed. This study is the first to evaluate various methods for diagnostic accuracy. In addition, this study has reviewed 18 articles (Ref. [17, 18, 19, 20, 21, 22, 23, 24, 25,26, 27, 28, 29, 30, 31, 32, 34, 35]) and 4102 patients, which provides an excellent reference value for clinical application.

The limitations are as follows. First, most of the selected researches performed TEE and another test method within one week, but one study completed both tests within one month. LAA/LAA thrombus may have formed/ dissolved between the two examinations. Second, the sample sizes of some studies were small. Some patients have been excluded due to examination contraindications, which may have affected the prevalence of LAA/LAA thrombus. Hopefully, there will be more studies to increase the sample size of different diagnostic methods to further verify the accuracy of the test.

## 6. Conclusions

This study included 10 diagnostic methods: 2D-CMRI, 3D-CMRI, CCTA, MDCT, DSCT, non-delayed CCT, 1-minute delayed CCT, 3-minute delayed CCT, 6-minute delayed CCT, and 3D-TEE; and the results showed that DSCT is superior to TEE in the sensitivity, specificity, NLR, and accuracy to determine thrombus of LA/LAA in AF patients. The outcomes will need to be substantiated by further research.

## Data Availability

The datasets for this study can be found in the article/**Supplementary Material**.

## Supplementary Material

Supplementary material associated with this article can be found, in the online version, at https://doi.org/10.31083/j.rcm2411334.
