# Supplementary material for: Diagnostic Accuracy of Left Atrial/Left Atrial Appendage Thrombus in Patients with Atrial Fibrillation: A Systematic Review and Network Meta-Analysis
Source: Rev Cardiovasc Med. 2023 Nov 27;24(11):334. doi: 10.31083/j.rcm2411334 (PMC11272870; doi:10.31083/j.rcm2411334)
Supplement: Supplementary file 1 [file 2153-8174-24-11-334-s1.zip › 2153-8174-24-11-334-s1/Supplementary Material-V2 for reviewing..docx]

*Systematic Review*

Diagnostic accuracy of left atrial/left atrial appendage thrombus in patients with atrial fibrillation:a systematic review and network meta-analysis

Supplementary Table 1. Search strategy on PubMed.

| Search | Query |  |
| --- | --- | --- |
| #1 | **(Atrial fibrillation[MeSH Terms]) OR (Atrial Fibrillations[Title/Abstract] OR Fibrillation, Atrial[Title/Abstract] OR Fibrillations, Atrial[Title/Abstract] OR Auricular Fibrillations[Title/Abstract] OR Auricular Fibrillation[Title/Abstract] OR Fibrillation, Auricular[Title/Abstract] OR Fibrillations, Auricular[Title/Abstract] OR Persistent Atrial Fibrillation[Title/Abstract] OR Atrial Fibrillation, Persistent[Title/Abstract] OR Atrial Fibrillations, Persistent[Title/Abstract] OR Fibrillation, Persistent Atrial[Title/Abstract] OR Fibrillations, Persistent Atrial[Title/Abstract] OR Persistent Atrial Fibrillations[Title/Abstract] OR Familial Atrial Fibrillation[Title/Abstract] OR Atrial Fibrillation, Familial[Title/Abstract] OR Familial Atrial Fibrillations[Title/Abstract] OR Atrial Fibrillations, Familial[Title/Abstract] OR Fibrillation, Familial Atrial[Title/Abstract] OR Fibrillations, Familial Atrial[Title/Abstract] OR Paroxysmal Atrial Fibrillation[Title/Abstract] OR Atril Fibrillation, Paroxysmal[Title/Abstract] OR Atrial Fibrillations, Paroxysmal[Title/Abstract] OR Fibrillation, Paroxysmal Atrial[Title/Abstract] OR Fibrillations, Paroxysmal Atrial[Title/Abstract] OR Paroxysmal Atrial Fibrillations[Title/Abstract])** | 70490 |
| #2 | **(left atrial appendage thrombus[Title/Abstract]) OR (Left atrial thrombus[Title/Abstract])** | **1282** |
| #3 | **(Echocardiography, Transesophageal[MeSH Terms]) OR (Transesophageal echocardiography[Title/Abstract])** | 28687 |
| #4 | **((Transthoracic echocardiography[MeSH Terms]) ) OR (Transthoracic Echocardiography[Title/Abstract] OR Echocardiography, Cross-Sectional[Title/Abstract] OR Echocardiography, Transthoracic[Title/Abstract] OR Echocardiography, Cross Sectional[Title/Abstract] OR Cross-Sectional Echocardiography[Title/Abstract] OR Cross Sectional Echocardiography[Title/Abstract] OR Echocardiography, M-Mode[Title/Abstract] OR Echocardiography, M Mode[Title/Abstract] OR M-Mode Echocardiography[Title/Abstract] OR M Mode Echocardiography[Title/Abstract] OR Echocardiography, Contrast[Title/Abstract] OR Contrast Echocardiography[Title/Abstract] OR 2D Echocardiography[Title/Abstract] OR Echocardiography, Two-Dimensional[Title/Abstract] OR Echocardiography, Two Dimensional[Title/Abstract] OR Echocardiography, 2D[Title/Abstract] OR Echocardiography, 2-D[Title/Abstract] OR Echocardiography, 2 D[Title/Abstract] OR Two-Dimensional Echocardiography[Title/Abstract] OR Two Dimensional Echocardiography[Title/Abstract] OR 2-D Echocardiography[Title/Abstract] OR 2 D Echocardiography[Title/Abstract])** | **155534** |
| #5 | **(Positron Emission Tomography Computed Tomography[MeSH Terms]) OR (PET-CT Scans[Title/Abstract] OR Scan, PET-CT[Title/Abstract] OR Scans, PET-CT[Title/Abstract] OR PET CT Scan[Title/Abstract] OR CT Scan, PET[Title/Abstract] OR CT Scans, PET[Title/Abstract] OR PET CT Scans[Title/Abstract] OR Scan, PET CT[Title/Abstract] OR Scans, PET CT[Title/Abstract] OR CT PET[Title/Abstract] OR Positron Emission Tomography-Computed Tomography[Title/Abstract] OR PET-CT[Title/Abstract] OR CT PET Scan[Title/Abstract] OR CT PET Scans[Title/Abstract] OR PET Scan, CT[Title/Abstract] OR PET Scans, CT[Title/Abstract] OR Scan, CT PET[Title/Abstract] OR Scans, CT PET[Title/Abstract])** | 42705 |
| #6 | **Multiplane transesophageal echocardiography[Title/Abstract] OR Three dimensional transesophageal echocardiography[Title/Abstract] OR Transesophageal contrast-enhanced ultrasound[Title/Abstract] OR Three dimensional transthoracic echocardiography[Title/Abstract] OR Cardiac computed tomography[Title/Abstract] OR Multi slice spiral enhanced CT Dual phase enhanced CT scanning[Title/Abstract] OR Double source 256 slice CT[Title/Abstract] OR Cardiac magnetic resonance[Title/Abstract] OR Pulmonary vein CTA[Title/Abstract] OR Multi slice spiral CT[Title/Abstract]** | **17847** |
| #7 | **#3 OR #4 OR #5 OR #6** | **214601** |
| #8 | **#1 AND #2 AND #7** | **401** |

Supplementary Table S1. Consistency test for Se.

|  | Coef. Std. Err z P>z [95% Conf. Interval] |
| --- | --- |
| B VS cons | -.4602918 .3636238 -1.27 0.206 -1.172981 .2523977 |
| CVS cons | .0069733 .1759523 0.04 0.968 -.337887 .3518335 |
| D VS cons | -.5802921 .3613931 -1.61 0.108 -1.28861 .1280254 |
| E VS cons | .0697042 .320857 0.22 0.828 -.5591639 .6985723 |
| F VS cons | -.0120599 .2301004 -0.05 0.958 -.4630483 .4389286 |
| G VS cons | .0697042 .3279145 0.21 0.832 -.5729964 .7124047 |
| H VS cons | .0697043 .2365403 0.29 0.768 -.3939062 .5333148 |
| I VS cons | -.0467742 .1796675 -0.26 0.795 -.3989161 .3053677 |
| J VS cons | .0697045 .1507826 0.46 0.644 -.225824 .3652331 |
| K VS cons | -.1249173 .1682027 -0.74 0.458 -.4545884 .2047539 |

Supplementary Table S2. Consistency test for Sp

|  | Coef. Std. Err z P>z [95% Conf. Interval] |
| --- | --- |
| B VS cons | -.4700753 .2239719 -2.10 0.036 -.9090522 -.0310985 |
| CVS cons | .0128791 .0104357 1.23 0.217 -.0075744 .0333326 |
| D VS cons | -.3000756 .2260877 -1.33 0.184 -.7431992 .1430481 |
| E VS cons | .0199224 .105218 0.19 0.850 -.1863012 .2261459 |
| F VS cons | .0222246 .0625995 0.36 0.723 -.1004681 .1449173 |
| G VS cons | -.1900774 .1220402 -1.56 0.119 -.4292718 .049117 |
| H VS cons | -.0829853 .0510116 -1.63 0.104 -.1829661 .0169956 |
| I VS cons | -.0344243 .0429083 -0.80 0.422 -.118523 .0496744 |
| J VS cons | .0299224 .0368688 0.81 0.417 -.0423392 .1021839 |
| K VS cons | -.1825451 .0227536 -8.02 0.000 -.2271413 -.1379489 |

Supplementary Table S3. Consistency test for PLR

|  | Coef. Std. Err z P>z [95% Conf. Interval] |
| --- | --- |
| B VS cons | -5.723322 7.404355 -0.77 0.440 -20.23559 8.788946 |
| CVS cons | -9.182378 5.952393 -1.54 0.123 -20.84885 2.484099 |
| D VS cons | -5.523324 7.404486 -0.75 0.456 -20.03585 8.989201 |
| E VS cons | -5.623522 7.402511 -0.76 0.447 -20.13218 8.885134 |
| F VS cons | -7.640946 6.749941 -1.13 0.258 -20.87059 5.588694 |
| G VS cons | -2.129792 7.45879 -0.29 0.775 -16.74875 12.48917 |
| H VS cons | 2.167103 6.092129 0.36 0.722 -9.77325 14.10746 |
| I VS cons | 2.701307 4.797104 0.56 0.573 -6.700843 12.10346 |
| J VS cons | -5.633901 4.236194 -1.33 0.184 -13.93669 2.668887 |
| K VS cons | -1.960871 4.680991 -0.42 0.675 -11.13545 7.213703 |

Supplementary Table S4. Consistency test for NLR

|  | Coef. Std. Err z P>z [95% Conf. Interval] |
| --- | --- |
| B VS cons | .964511 .4663236 2.07 0.039 .0505335 1.878488 |
| CVS cons | -.0101794 .2468739 -0.04 0.967 -.4940433 .4736846 |
| D VS cons | .8745113 .4645161 1.88 0.060 -.0359234 1.784946 |
| E VS cons | -.0954775 .4362016 -0.22 0.827 -.950417 .7594619 |
| F VS cons | .015162 .3224304 0.05 0.962 -.6167899 .647114 |
| G VS cons | -.0954775 .4414187 -0.22 0.829 -.9606422 .7696872 |
| H VS cons | .4131889 .3314454 1.25 0.213 -.2364321 1.06281 |
| I VS cons | .0191262 .2471871 0.08 0.938 -.4653517 .503604 |
| J VS cons | .9045217 .2076859 4.36 0.000 .4974648 1.311579 |
| K VS cons | .1663057 .2291861 0.73 0.468 -.2828908 .6155023 |

Supplementary Table S5. Consistency test for Accuracy

|  | Coef. Std. Err z P>z [95% Conf. Interval] |
| --- | --- |
| B VS cons | -.8806165 .3240192 -2.72 0.007 -1.515682 -.2455505 |
| CVS cons | .0420138 .1465228 0.29 0.774 -.2451656 .3291932 |
| D VS cons | -.8906165 .3239857 -2.75 0.006 -1.525617 -.2556162 |
| E VS cons | .0793768 .2668015 0.30 0.766 -.4435445 .6022981 |
| F VS cons | .0297513 .1975212 0.15 0.880 -.3573831 .4168857 |
| G VS cons | -.1306225 .2738722 -0.48 0.633 -.6674022 .4061571 |
| H VS cons | .0310081 .2032744 0.15 0.879 -.3674024 .4294186 |
| I VS cons | -.1112115 .1533059 -0.73 0.468 -.4116856 .1892625 |
| J VS cons | .0893771 .1286474 0.69 0.487 -.1627672 .3415213 |
| K VS cons | -.2450698 .1383638 -1.77 0.077 -.5162578 .0261183 |

Supplementary Table S6.League table on Se

| _DSCT_ | _3-minute delayed CCT_ | _1-minute delayed CCT_ | _MDCT_ | _3D-CMR_ | _TEE_ | _non-delayed CCT_ | _6-minute delayed CCT_ | _3D-TEE_ | _2D-CMR_ | _CCTA_ |
| --- | --- | --- | --- | --- | --- | --- | --- | --- | --- | --- |
| DSCT | -0.00(-0.36,0.36) | -0.00(-0.57,0.57) | -0.00 (-0.56,0.56) | -0.06 (-0.40,0.27) | -0.07 (-0.37,0.23) | -0.08 (-0.52,0.36) | -0.12 (-0.31,0.07) | -0.19 (-0.47,0.08) | -0.53 (-1.18,0.12) | -0.65 (-1.29,-0.01) |
| 0.00 (-0.36,0.36) | 3-minute-delayed-CCT | -0.00 (-0.67,0.67) | -0.00 (-0.66,0.66) | -0.06 (-0.55,0.43) | -0.07 (-0.53,0.39) | -0.08 (-0.65,0.49) | -0.12 (-0.52,0.29) | -0.19 (-0.65,0.26) | -0.53 (-1.27,0.21) | -0.65 (-1.39,0.09) |
| 0.00 (-0.57,0.57) | 0.00 (-0.67,0.67) | 1-minute-delayed-CCT | 0.00 (-0.80,0.80) | -0.06 (-0.73,0.60) | -0.07 (-0.71,0.57) | -0.08 (-0.80,0.64) | -0.12 (-0.72,0.49) | -0.19 (-0.83,0.44) | -0.53 (-1.39,0.33) | -0.65 (-1.51,0.21) |
| 0.00 (-0.56,0.56) | 0.00 (-0.66,0.66) | -0.00 (-0.80,0.80) | MDCT | -0.06 (-0.71,0.59) | -0.07 (-0.70,0.56) | -0.08 (-0.79,0.63) | -0.12 (-0.70,0.47) | -0.19 (-0.81,0.42) | -0.53 (-1.38,0.32) | -0.65 (-1.50,0.20) |
| 0.06 (-0.27,0.40) | 0.06 (-0.43,0.55) | 0.06 (-0.60,0.73) | 0.06 (-0.59,0.71) | 3D-CMR | -0.01 (-0.35,0.34) | -0.02 (-0.49,0.45) | -0.05 (-0.44,0.33) | -0.13 (-0.51,0.24) | -0.47 (-1.20,0.26) | -0.59 (-1.31,0.14) |
| 0.07 (-0.23,0.37) | 0.07 (-0.39,0.53) | 0.07 (-0.57,0.71) | 0.07 (-0.56,0.70) | 0.01 (-0.34,0.35) | TEE | -0.01 (-0.46,0.44) | -0.05 (-0.40,0.31) | -0.12 (-0.45,0.20) | -0.46 (-1.17,0.25) | -0.58 (-1.29,0.13) |
| 0.08 (-0.36,0.52) | 0.08 (-0.49,0.65) | 0.08 (-0.64,0.80) | 0.08 (-0.63,0.79) | 0.02 (-0.45,0.49) | 0.01 (-0.44,0.46) | non-delayed-CCT | -0.03 (-0.51,0.45) | -0.11 (-0.57,0.34) | -0.45 (-1.23,0.34) | -0.57 (-1.35,0.21) |
| 0.12 (-0.07,0.31) | 0.12 (-0.29,0.52) | 0.12 (-0.49,0.72) | 0.12 (-0.47,0.70) | 0.05 (-0.33,0.44) | 0.05 (-0.31,0.40) | 0.03 (-0.45,0.51) | 6-minute-delayed-CCT | -0.08 (-0.41,0.26) | -0.41 (-1.09,0.26) | -0.53 (-1.20,0.14) |
| 0.19 (-0.08,0.47) | 0.19 (-0.26,0.65) | 0.19 (-0.44,0.83) | 0.19 (-0.42,0.81) | 0.13 (-0.24,0.51) | 0.12 (-0.20,0.45) | 0.11 (-0.34,0.57) | 0.08 (-0.26,0.41) | 3D-TEE | -0.34 (-1.04,0.37) | -0.46 (-1.16,0.24) |
| 0.53 (-0.12,1.18) | 0.53 (-0.21,1.27) | 0.53 (-0.33,1.39) | 0.53 (-0.32,1.38) | 0.47 (-0.26,1.20) | 0.46 (-0.25,1.17) | 0.45 (-0.34,1.23) | 0.41 (-0.26,1.09) | 0.34 (-0.37,1.04) | 2D-CMR | -0.12 (-0.64,0.40) |
| 0.65 (0.01,1.29) | 0.65 (-0.09,1.39) | 0.65 (-0.21,1.51) | 0.65 (-0.20,1.50) | 0.59 (-0.14,1.31) | 0.58 (-0.13,1.29) | 0.57 (-0.21,1.35) | 0.53 (-0.14,1.20) | 0.46 (-0.24,1.16) | 0.12 (-0.40,0.64) | CCTA |

Supplementary Table S7. League table on Sp

| _DSCT_ | __3D_CMR_ | _non_delayed_CCT_ | _MDCT_ | _TEE_ | __6_minute_delayed_CCT_ | __3_minute_delayed_CCT_ | __1_minute_delayed_CCT_ | _CCTA_ | __3D_TEE_ | __2D_CMR_ |
| --- | --- | --- | --- | --- | --- | --- | --- | --- | --- | --- |
| DSCT | -0.02 (-0.09,0.06) | -0.01  (-0.15,0.13) | -0.01 (-0.20,0.18) | -0.03 (-0.10,0.04) | -0.06  (-0.11,-0.02) | -0.11  (-0.18,-0.04) | -0.22  (-0.45,0.01) | -0.33  (-0.77,0.11) | -0.21 (-0.29,-0.13) | -0.50 (-0.93,-0.07) |
| 0.02 (-0.06,0.09) | 3D-CMR | 0.01  (-0.11,0.13) | 0.01 (-0.20,0.21) | -0.01 (-0.03,0.01) | -0.05  (-0.13,0.04) | -0.10  (-0.20,0.00) | -0.20  (-0.44,0.04) | -0.31  (-0.76,0.13) | -0.20 (-0.24,-0.15) | -0.48 (-0.92,-0.04) |
| 0.01 (-0.13,0.15) | -0.01 (-0.13,0.11) | non-delayed-CCT | -0.00 (-0.24,0.24) | -0.02 (-0.14,0.10) | -0.06  (-0.20,0.09) | -0.11  (-0.26,0.05) | -0.21  (-0.48,0.06) | -0.32  (-0.78,0.14) | -0.20 (-0.33,-0.08) | -0.49 (-0.95,-0.04) |
| 0.01 (-0.18,0.20) | -0.01 (-0.21,0.20) | 0.00  (-0.24,0.24) | MDCT | -0.02 (-0.23,0.19) | -0.05  (-0.25,0.14) | -0.10  (-0.31,0.10) | -0.21  (-0.51,0.09) | -0.32  (-0.80,0.16) | -0.20 (-0.41,0.01) | -0.49 (-0.96,-0.02) |
| 0.03 (-0.04,0.10) | 0.01 (-0.01,0.03) | 0.02  (-0.10,0.14) | 0.02 (-0.19,0.23) | TEE | -0.03  (-0.12,0.05) | -0.08  (-0.18,0.02) | -0.19  (-0.43,0.05) | -0.30  (-0.74,0.14) | -0.18 (-0.23,-0.14) | -0.47 (-0.91,-0.03) |
| 0.06  (0.02,0.11) | 0.05 (-0.04,0.13) | 0.06  (-0.09,0.20) | 0.05 (-0.14,0.25) | 0.03  (-0.05,0.12) | 6-minute-delayed-CCT | -0.05  (-0.13,0.03) | -0.16  (-0.39,0.08) | -0.27  (-0.70,0.17) | -0.15 (-0.24,-0.06) | -0.44 (-0.87,-0.00) |
| 0.11  (0.04,0.18) | 0.10 (-0.00,0.20) | 0.11  (-0.05,0.26) | 0.10 (-0.10,0.31) | 0.08  (-0.02,0.18) | 0.05  (-0.03,0.13) | 3-minute-delayed-CCT | -0.11  (-0.35,0.13) | -0.22  (-0.66,0.23) | -0.10 (-0.20,0.00) | -0.39 (-0.83,0.05) |
| 0.22 (-0.01,0.45) | 0.20 (-0.04,0.44) | 0.21  (-0.06,0.48) | 0.21 (-0.09,0.51) | 0.19  (-0.05,0.43) | 0.16  (-0.08,0.39) | 0.11  (-0.13,0.35) | 1-minute-delayed-CCT | -0.11  (-0.60,0.38) | 0.01 (-0.23,0.25) | -0.28 (-0.77,0.21) |
| 0.33 (-0.11,0.77) | 0.31 (-0.13,0.76) | 0.32  (-0.14,0.78) | 0.32 (-0.16,0.80) | 0.30  (-0.14,0.74) | 0.27  (-0.17,0.70) | 0.22  (-0.23,0.66) | 0.11  (-0.38,0.60) | CCTA | 0.12 (-0.33,0.56) | -0.17 (-0.38,0.04) |
| 0.21 (0.13,0.29) | 0.20 (0.15,0.24) | 0.20  (0.08,0.33) | 0.20 (-0.01,0.41) | 0.18  (0.14,0.23) | 0.15  (0.06,0.24) | 0.10  (-0.00,0.20) | -0.01  (-0.25,0.23) | -0.12  (-0.56,0.33) | 3D-TEE | -0.29 (-0.73,0.15) |
| 0.50  (0.07,0.93) | 0.48 (0.04,0.92) | 0.49  (0.04,0.95) | 0.49 (0.02,0.96) | 0.47  (0.03,0.91) | 0.44  (0.00,0.87) | 0.39  (-0.05,0.83) | 0.28  (-0.21,0.77) | 0.17  (-0.04,0.38) | 0.29 (-0.15,0.73) | 2D-CMR |

Supplementary Table S8.League table on PLR

| __6_minute_delayed_CCT_ | __3_minute_delayed_CCT_ | _TEE_ | __3D_TEE_ | __1_minute_delayed_CCT_ | _MDCT_ | _CCTA_ | __2D_CMR_ | _DSCT_ | _non_delayed_CCT_ | __3D_CMR_ |
| --- | --- | --- | --- | --- | --- | --- | --- | --- | --- | --- |
| 6-minute-delayed-CCT | -0.53 (-10.35,9.28) | -2.70 (-12.10,6.70) | -4.66 (-12.27,2.95) | -4.83 (-17.78,8.11) | -8.32 (-21.14,4.49) | -8.22 (-21.05,4.60) | -8.42 (-21.25,4.40) | -8.34 (-13.08,-3.59) | -10.34 (-22.14,1.45) | -11.88 (-23.93,0.16) |
| 0.53 (-9.28,10.35) | 3-minute-delayed-CCT | -2.17 (-14.11,9.77) | -4.13 (-14.58,6.33) | -4.30 (-19.09,10.50) | -7.79 (-22.48,6.90) | -7.69 (-22.38,7.00) | -7.89 (-22.58,6.80) | -7.80 (-16.40,0.79) | -9.81 (-23.56,3.95) | -11.35 (-25.41,2.71) |
| 2.70 (-6.70,12.10) | 2.17 (-9.77,14.11) | TEE | -1.96 (-11.14,7.21) | -2.13 (-16.75,12.49) | -5.62 (-20.13,8.89) | -5.52 (-20.04,8.99) | -5.72 (-20.24,8.79) | -5.63 (-13.94,2.67) | -7.64 (-20.87,5.59) | -9.18 (-20.85,2.48) |
| 4.66 (-2.95,12.27) | 4.13 (-6.33,14.58) | 1.96 (-7.21,11.14) | 3D-TEE | -0.17 (-13.60,13.27) | -3.66 (-16.98,9.65) | -3.56 (-16.88,9.76) | -3.76 (-17.08,9.56) | -3.67 (-9.63,2.28) | -5.68 (-16.45,5.09) | -7.22 (-19.50,5.06) |
| 4.83 (-8.11,17.78) | 4.30 (-10.50,19.09) | 2.13 (-12.49,16.75) | 0.17 (-13.27,13.60) | 1-minute-delayed-CCT | -3.49 (-20.43,13.44) | -3.39 (-20.34,13.55) | -3.59 (-20.54,13.35) | -3.50 (-15.55,8.54) | -5.51 (-21.65,10.63) | -7.05 (-23.45,9.34) |
| 8.32 (-4.49,21.14) | 7.79 (-6.90,22.48) | 5.62 (-8.89,20.13) | 3.66 (-9.65,16.98) | 3.49 (-13.44,20.43) | MDCT | 0.10 (-16.75,16.95) | -0.10 (-16.95,16.75) | -0.01 (-11.92,11.90) | -2.02 (-18.06,14.02) | -3.56 (-19.85,12.74) |
| 8.22 (-4.60,21.05) | 7.69 (-7.00,22.38) | 5.52 (-8.99,20.04) | 3.56 (-9.76,16.88) | 3.39 (-13.55,20.34) | -0.10 (-16.95,16.75) | CCTA | -0.20 (-12.11,11.71) | -0.11 (-12.03,11.80) | -2.12 (-18.16,13.92) | -3.66 (-19.96,12.64) |
| 8.42 (-4.40,21.25) | 7.89 (-6.80,22.58) | 5.72 (-8.79,20.24) | 3.76 (-9.56,17.08) | 3.59 (-13.35,20.54) | 0.10 (-16.75,16.95) | 0.20 (-11.71,12.11) | 2D-CMR | 0.09 (-11.83,12.00) | -1.92 (-17.96,14.12) | -3.46 (-19.76,12.84) |
| 8.34 (3.59,13.08) | 7.80 (-0.79,16.40) | 5.63 (-2.67,13.94) | 3.67 (-2.28,9.63) | 3.50 (-8.54,15.55) | 0.01 (-11.90,11.92) | 0.11 (-11.80,12.03) | -0.09 (-12.00,11.83) | DSCT | -2.01 (-12.75,8.73) | -3.55 (-14.67,7.58) |
| 10.34 (-1.45,22.14) | 9.81 (-3.95,23.56) | 7.64 (-5.59,20.87) | 5.68 (-5.09,16.45) | 5.51 (-10.63,21.65) | 2.02 (-14.02,18.06) | 2.12 (-13.92,18.16) | 1.92 (-14.12,17.96) | 2.01 (-8.73,12.75) | non-delayed-CCT | -1.54 (-16.87,13.79) |
| 11.88 (-0.16,23.93) | 11.35 (-2.71,25.41) | 9.18 (-2.48,20.85) | 7.22 (-5.06,19.50) | 7.05 (-9.34,23.45) | 3.56 (-12.74,19.85) | 3.66 (-12.64,19.96) | 3.46 (-12.84,19.76) | 3.55 (-7.58,14.67) | 1.54 (-13.79,16.87) | 3D-CMR |

Supplementary Table S9. League table on NLR

| _DSCT_ | __2D_CMR_ | _CCTA_ | __3_minute_delayed_CCT_ | __3D_TEE_ | _non_delayed_CCT_ | __6_minute_delayed_CCT_ | _TEE_ | __3D_CMR_ | _MDCT_ | __1_minute_delayed_CCT_ |
| --- | --- | --- | --- | --- | --- | --- | --- | --- | --- | --- |
| DSCT | 0.06 (-0.76,0.88) | -0.03  (-0.84,0.78) | -0.49  (-1.00,0.01) | -0.74 (-1.10,-0.37) | -0.89 (-1.51,-0.27) | -0.89  (-1.15,-0.62) | -0.90 (-1.31,-0.50) | -0.91 (-1.39,-0.44) | -1.00  (-1.75,-0.25) | -1.00  (-1.76,-0.24) |
| -0.06 (-0.88,0.76) | 2D-CMR | -0.09  (-0.80,0.62) | -0.55  (-1.51,0.41) | -0.80 (-1.69,0.10) | -0.95  (-1.97,0.07) | -0.95  (-1.80,-0.09) | -0.96 (-1.88,-0.05) | -0.97 (-1.92,-0.03) | -1.06  (-2.17,0.05) | -1.06  (-2.18,0.06) |
| 0.03 (-0.78,0.84) | 0.09 (-0.62,0.80) | CCTA | -0.46  (-1.42,0.50) | -0.71 (-1.60,0.18) | -0.86  (-1.88,0.16) | -0.86  (-1.71,0.00) | -0.87 (-1.78,0.04) | -0.88 (-1.83,0.06) | -0.97  (-2.08,0.14) | -0.97 (-2.09,0.15) |
| 0.49 (-0.01,1.00) | 0.55 (-0.41,1.51) | 0.46  (-0.50,1.42) | 3-minute-delayed-CCT | -0.25 (-0.87,0.38) | -0.40  (-1.20,0.40) | -0.39  (-0.96,0.18) | -0.41 (-1.06,0.24) | -0.42 (-1.12,0.27) | -0.51  (-1.42,0.40) | -0.51  (-1.42,0.41) |
| 0.74 (0.37,1.10) | 0.80 (-0.10,1.69) | 0.71  (-0.18,1.60) | 0.25  (-0.38,0.87) | 3D-TEE | -0.15  (-0.78,0.48) | -0.15  (-0.60,0.30) | -0.17 (-0.62,0.28) | -0.18 (-0.69,0.34) | -0.26  (-1.10,0.57) | -0.26  (-1.11,0.58) |
| 0.89 (0.27,1.51) | 0.95 (-0.07,1.97) | 0.86  (-0.16,1.88) | 0.40  (-0.40,1.20) | 0.15 (-0.48,0.78) | non-delayed-CCT | 0.00  (-0.67,0.67) | -0.02 (-0.65,0.62) | -0.03 (-0.68,0.63) | -0.11  (-1.08,0.86) | -0.11  (-1.09,0.87) |
| 0.89 (0.62,1.15) | 0.95 (0.09,1.80) | 0.86  (-0.00,1.71) | 0.39  (-0.18,0.96) | 0.15 (-0.30,0.60) | -0.00  (-0.67,0.67) | 6-minute-delayed-CCT | -0.02 (-0.50,0.47) | -0.03 (-0.57,0.51) | -0.11  (-0.91,0.68) | -0.11  (-0.92,0.69) |
| 0.90 (0.50,1.31) | 0.96 (0.05,1.88) | 0.87  (-0.04,1.78) | 0.41  (-0.24,1.06) | 0.17 (-0.28,0.62) | 0.02  (-0.62,0.65) | 0.02  (-0.47,0.50) | TEE | -0.01 (-0.49,0.47) | -0.10  (-0.95,0.76) | -0.10  (-0.96,0.77) |
| 0.91 (0.44,1.39) | 0.97 (0.03,1.92) | 0.88  (-0.06,1.83) | 0.42  (-0.27,1.12) | 0.18 (-0.34,0.69) | 0.03  (-0.63,0.68) | 0.03  (-0.51,0.57) | 0.01 (-0.47,0.49) | 3D-CMR | -0.09  (-0.97,0.80) | -0.09  (-0.98,0.81) |
| 1.00 (0.25,1.75) | 1.06 (-0.05,2.17) | 0.97  (-0.14,2.08) | 0.51  (-0.40,1.42) | 0.26 (-0.57,1.10) | 0.11  (-0.86,1.08) | 0.11  (-0.68,0.91) | 0.10 (-0.76,0.95) | 0.09 (-0.80,0.97) | MDCT | 0.00  (-1.07,1.07) |
| 1.00 (0.24,1.76) | 1.06 (-0.06,2.18) | 0.97  (-0.15,2.09) | 0.51  (-0.41,1.42) | 0.26 (-0.58,1.11) | 0.11  (-0.87,1.09) | 0.11  (-0.69,0.92) | 0.10 (-0.77,0.96) | 0.09 (-0.81,0.98) | -0.00  (-1.07,1.07) | 1-minute-delayed-CCT |

Supplementary Table. S10 League table on Accuracy

| _DSCT_ | _MDCT_ | __3D_CMR_ | __3_minute_delayed_CCT_ | _non_delayed_CCT_ | _TEE_ | __1_minute_delayed_CCT_ | __6_minute_delayed_CCT_ | __3D_TEE_ | __2D_CMR_ | _CCTA_ |
| --- | --- | --- | --- | --- | --- | --- | --- | --- | --- | --- |
| DSCT | -0.01 (-0.47,0.45) | -0.05 (-0.33,0.24) | -0.06  (-0.37,0.25) | -0.06 (-0.44,0.32) | -0.09 (-0.34,0.16) | -0.22  (-0.69,0.25) | -0.20  (-0.36,-0.04) | -0.33 (-0.56,-0.11) | -0.97 (-1.55,-0.39) | -0.98 (-1.56,-0.40) |
| 0.01 (-0.45,0.47) | MDCT | -0.04 (-0.58,0.50) | -0.05  (-0.60,0.50) | -0.05 (-0.65,0.55) | -0.08 (-0.60,0.44) | -0.21  (-0.87,0.45) | -0.19  (-0.68,0.30) | -0.32 (-0.84,0.19) | -0.96 (-1.70,-0.22) | -0.97 (-1.71,-0.23) |
| 0.05 (-0.24,0.33) | 0.04  (-0.50,0.58) | 3D-CMR | -0.01  (-0.43,0.41) | -0.01 (-0.41,0.39) | -0.04 (-0.33,0.25) | -0.17  (-0.73,0.38) | -0.1  (-0.48,0.18) | -0.29 (-0.60,0.02) | -0.92 (-1.57,-0.27) | -0.93 (-1.58,-0.28) |
| 0.06 (-0.25,0.37) | 0.05  (-0.50,0.60) | 0.01  (-0.41,0.43) | 3-minute-delayed-CCT | -0.00 (-0.49,0.49) | -0.03 (-0.43,0.37) | -0.16  (-0.73,0.40) | -0.14 (-0.49,0.21) | -0.28 (-0.66,0.11) | -0.91 (-1.57,-0.25) | -0.92 (-1.58,-0.26) |
| 0.06 (-0.32,0.44) | 0.05  (-0.55,0.65) | 0.01  (-0.39,0.41) | 0.00  (-0.49,0.49) | non-delayed-CCT | -0.03 (-0.42,0.36) | -0.16  (-0.77,0.45) | -0.14  (-0.56,0.27) | -0.27 (-0.66,0.11) | -0.91 (-1.61,-0.21) | -0.92 (-1.62,-0.22) |
| 0.09 (-0.16,0.34) | 0.08  (-0.44,0.60) | 0.04  (-0.25,0.33) | 0.03  (-0.37,0.43) | 0.03 (-0.36,0.42) | TEE | -0.13  (-0.67,0.41) | -0.11  (-0.41,0.19) | -0.25 (-0.52,0.03) | -0.88 (-1.52,-0.25) | -0.89 (-1.53,-0.26) |
| 0.22 (-0.25,0.69) | 0.21  (-0.45,0.87) | 0.17  (-0.38,0.73) | 0.16  (-0.40,0.73) | 0.16 (-0.45,0.77) | 0.13 (-0.41,0.67) | 1-minute-delayed-CCT | 0.02  (-0.48,0.52) | -0.11 (-0.64,0.41) | -0.75 (-1.50,0.00) | -0.76 (-1.51,-0.01) |
| 0.20 (0.04,0.36) | 0.19  (-0.30,0.68) | 0.15  (-0.18,0.48) | 0.14  (-0.21,0.49) | 0.14 (-0.27,0.56) | 0.11 (-0.19,0.41) | -0.02  (-0.52,0.48) | 6-minute-delayed-CCT | -0.13 (-0.41,0.15) | -0.77 (-1.37,-0.16) | -0.78 (-1.38,-0.17) |
| 0.33 (0.11,0.56) | 0.32  (-0.19,0.84) | 0.29  (-0.02,0.60) | 0.28  (-0.11,0.66) | 0.27 (-0.11,0.66) | 0.25 (-0.03,0.52) | 0.11  (-0.41,0.64) | 0.13  (-0.15,0.41) | 3D-TEE | -0.64 (-1.26,-0.01) | -0.65 (-1.27,-0.02) |
| 0.97 (0.39,1.55) | 0.96  (0.22,1.70) | 0.92  (0.27,1.57) | 0.91  (0.25,1.57) | 0.91 (0.21,1.61) | 0.88 (0.25,1.52) | 0.75  (-0.00,1.50) | 0.77  (0.16,1.37) | 0.64 (0.01,1.26) | 2D-CMR | -0.01 (-0.43,0.41) |
| 0.98 (0.40,1.56) | 0.97  (0.23,1.71) | 0.93  (0.28,1.58) | 0.92  (0.26,1.58) | 0.92 (0.22,1.62) | 0.89 (0.26,1.53) | 0.76  (0.01,1.51) | 0.78  (0.17,1.38) | 0.65 (0.02,1.27) | 0.01 (-0.41,0.43) | CCTA |
